# Improving Workplace-Based Learning for Undergraduate Medical Students

**DOI:** 10.12669/pjms.315.7687

**Published:** 2015

**Authors:** Madiha Sajjad, Usman Mahboob

**Affiliations:** 1 Madiha Sajjad, Associate Professor of Pathology, Islamic International Medical College, Riphah International University, Rawalpindi, Pakistan; 2 Usman Mahboob, Assistant Professor of Medical Education, Institute of Health Professions Education & Research, Khyber Medical University, Peshawar, Pakistan

**Keywords:** Workplace Based Learning, Undergraduate Medical Students

## Abstract

Workplace-based learning is considered as one of the most effective way of translating medical theory into clinical practice. Although employed traditionally at postgraduate level, this strategy can be used in undergraduate students coming for clerkships in clinical departments. There are many challenges to workplace learning such as, unfavorable physical environment, lack of interest by clinical staff and teachers, and lack of student motivation. Clinical teachers can help bridge this gap and improve workplace learning through individual and collaborative team effort. Knowledge of various educational theories and principles and their application at workplace can enhance student learning and motivation, for which faculty development is much needed. Different teaching and learning activities can be used and tailored according to the clinical setting. Active reflection by students and constructive feedback from the clinicians forms the backbone of effective workplace learning.

## INTRODUCTION

‘Workplace-based learning’, also termed as ‘situational learning’ may be defined as a learning experience which allows students to learn through supervised application of their professional roles in real workplace settings.^1^ It has its roots in the age old ‘apprenticeship model’ where the trainee students shadowed physicians for their clinical training.^2^,^3^ Abandoned initially due to lack of scientific reasoning at its base in favor of more formal college education, the pendulum has swung back to it with realization of the need for early exposure of undergraduate medical students to clinical environments but in a more structured manner.^2^,^4^ Hospitals, clinics and community provide the ideal platform, where students have an opportunity to apply knowledge to practice and develop competence. If properly planned, it can promote student motivation, help build confidence for patient interaction and inculcate in them a culture of reflection and self-appraisal.^4^,^5^ On the downside, there is added strain on the clinicians as educators. Time and priority constraints are a challenge. Additionally, they need to learn the currently used educational methods such as, student-centered teaching approach, competency-based assessment skills, professionalism and ethics to be able to efficiently balance all their roles.^5^–^7^

The various educational theories as applied to workplace learning are the ‘Experiential learning’,^2^,^6^ which emphasizes on learning as a cyclical process in which there is a connection between actual experience and abstract conceptualization through reflection and planning. Theories of cognition’ which hold that learning occurs by information processing in the mind. ‘Theories of constructivism’ that involves construction of new knowledge based on a previous knowledge, are a few important ones.^2^

Based on these theories various models of effective teaching and learning have emerged. For instance, the ‘FAIR model’ which is based on the four principles that students learn best when given proper ‘Feedback’ while ‘Actively’ participating in clinical activities, with attention to ‘Individualization’ and ‘Relevance’ to student needs.^4^,^7^ Moreover, the ‘One minute preceptor model’ can be used with modification in both clinical and laboratory environment using the same principle of; getting commitment on a diagnosis (for example on patient examination or laboratory report), probing for reasons behind this diagnosis, reinforcing the good points and guiding about omissions or errors and at the end, concluding with teaching the general principles (whether approach to patient examination or approach to a laboratory report or investigation).^2^,^8^ The key components of good workplace learning are thus ‘Supported participation’, i.e. deliberate matching of the student activities with their learning needs appropriate to their level of professional development and training.^4^ This should be coupled with ‘Constructive feedback’, which aims to correct, not insult and is timely, frequent, and non-evaluative.^3^,^4^,^7^

There are various challenges to workplace-based learning such as the physical learning environment, being under-resourced with lack of appropriate arrangement for student discussion,^2^ and the clinical staff perceiving students to be in the way of their assigned duties. Additionally, the clinicians involved in workplace teaching having limited knowledge of the educational principles so teaching may become didactic lecturing with more emphasis on factual knowledge rather than active student involvement and problem solving.^2^,^5^,^9^ Moreover, there are limitations like time constraints and conflict between the clinicians’ administrative duties and needs of students and patients.^2^–^5^

## IMPROVING WORKPLACE BASED LEARNING

Proper planning along with timely communication of learning outcomes, intended competencies and expected behaviors, is important for effective workplace learning.^2^,^4^ Clinical teachers can play a major role in this regard, as passionate and enthusiastic teachers are a major motivational factor for students. Appropriate time management and organization of work by the clinical teachers is mandatory.^5^ Moreover, explaining on the model of ‘loud thinking’, questioning, good listening abilities and choosing suitable clinical learning experiences for learners at different training levels, are the toolkit for good clinical teachers in developing sound clinical reasoning.^2^,^4^ Additionally, ensuring a conducive learning environment, making students feel respected, encouragement and praise for work well done, can all promote effective learning.^2^,^5^

Students role model the behaviors of clinicians with patients and staff imperceptibly to learn professionalism and right attitudes.^2^,^5^,^6^,^9^ Role plays can be used to overcome the issue of opportunistic learning and in teaching specific attitudes.^3^,^4^ One minute preceptor model can be used in ambulatory care settings.^5^ Simulations can be used in situations where patient safety is at risk.^5^ Active structured participation by students in day to day clinical activities is the key to learning in context.^4^,^8^

All activities need to be followed by reflection and feedback. Non-judgmental, timely and specific feedback, when given after direct monitoring of any learning activity is an effective workplace learning tool.^4^,^6^ Formative assessment is a means for better learning due to its connection with timely feedback.^10^

The experiential learning cycle advocates reflection as a major component in the learning process.^2^,^4^ Portfolios can be introduced as a learning tool to enhance reflective practices, in addition to their role in student assessment.^10^ Finally, teachers’ commitment is recognized by their ability to reflect on their own shortcomings, and getting feedback from students and colleagues. This is the essence of professional development with impact on improving quality of future workplace learning.^5^-^7^

In conclusion, the stakeholders involved directly in the workplace-based learning process include the students, clinicians and the patients supported by the clinical, administrative and other working staff.^5^,^6^ For effective learning, all of these need to be taken into consideration. Students are required to be active and reflective participants.^1^,^9^ Teachers on their part need to be enthusiastic, non-judgmental observers and listeners, giving appropriate clarifications and feedback where required. Patients need to be taken into confidence to be able to help in the process of learning, their privacy and safety being top priority in this respect.^4^

## Notes

Erratum to Pakistan Journal of Medical Sciences 2015 Assessment of the Frequency of Autoantibodies in Chronic Viral Hepatitis *Akif Acay^1^, Kasim Demir^2^, Gulsah Asik^3^, Havva Tunay^4^, Gursel Acarturk^5^*
The above manuscript was published in Pakistan Journal of Medical Sciences January-February 2015 Vol. 31 No. 1 Page NO. 150-154. The affiliation of one of the authors Havva Tunay was not mentioned correctly. His affiliation is Dept. of Infectious Diseases and Clinical Microbiology, Afyonkarahisar State Hospital, Afyonkarahisar. Turkey. Received for Publication: July 15, 2014 Revision Received: October 23, 2014 Revision Accepted: October 25, 2014
